# Effect of urgency level on prehospital emergency transport times: a natural experiment

**DOI:** 10.1007/s11739-023-03501-7

**Published:** 2023-12-20

**Authors:** Jan Brink Valentin, Nanna Høgh Hansen, Anne Brink Behrndtz, Ulla Væggemose, Martin Faurholdt Gude

**Affiliations:** 1 Danish Center for Health Services Research, Department of Clinical Medicine, Aalborg University, Aalborg, Denmark; 2 Department of Research and Development, Prehospital Emergency Medical Services, Central Denmark Region, Denmark; 3 Department of Neurology, Aarhus University Hospital, Aarhus, Denmark; 4 Department of Clinical Medicine, Aarhus University, Aarhus, Denmark

**Keywords:** Emergency medical services, EMS, Emergency services, Prehospital, Transportation of patients, Prehospital emergency care, Geographical information systems, Google Maps, Estimated time of arrival, Ambulances

## Abstract

Accurate estimation of ambulance transport time from the scene of incident to arrival at the emergency department (ED) is important for effective resource management and emergency care system planning. Further, differences in transport times between different urgency levels highlight the benefits of ambulance transports with highest urgency level in a setting where ambulances are allowed to not follow standard traffic rules. The objective of the study is to compare ambulance urgency level on the differences in estimates of ambulance transport times generated by Google Maps and the observed transport times in a prehospital setting where emergency vehicles have their own traffic laws. The study was designed as a natural experiment and register study. Ambulance transports dispatched with different levels of urgency (Level A and B) were included in the Central Denmark Region (a mixed urban and rural area) from March 10 to June 11, 2021. Ambulance transports for highest urgency level were compared to lowest urgency level with Google Maps estimated transport times as reference. We analyzed 1981 highest urgency level and 8.958 lowest urgency level ambulance transports. Google Maps significantly overestimated the duration of transports operating at highest level of urgency (Level A) by 1.9 min/10 km (95% CI 1.8; 2.0) in average and 4.8 min/10 km (95% CI 3.9; 5.6) for the first driven 10 km. Contrary, Google Maps significantly underestimated the duration of transports operating at lowest level of urgency (Level B) by −1.8 min/10 km (95% CI −2.1; −1.5) in average and −4.4 min/10 km (95% CI −5.4; −3.5) for the first driven 10 km. Google Maps systematically overestimates transport times of ambulance transports driven with Level A, the highest level of urgency in a setting where ambulances are allowed to not follow standard traffic rules. The results highlight the benefit of using urgency Level A and provide valuable information for emergency care management.

**Supplementary Information:**

The online version contains supplementary material available at 10.1007/s11739-023-03501-7.

## Introduction

Accurate estimation of ambulance transport time from the scene of accident or incident to arrival to the emergency department (ED) is important for effective resource management and emergency care system planning. Studies modeling prehospital transport often use estimates based on geographical information systems (GIS) that does not incorporate the time saved by highest level of urgency (Level A transportation) [1–8]. Factors that contribute to a fast transport when using highest urgency level include the use of lights and sirens (L&S), and the avoidance of normal rules for traffic regulation (e.g., exceeding the posted speed limit, proceeding through red traffic signal, stop light or stop sign, bypassing traffic jam using alternative routes, e.g., sidewalk, etc.).

Different attempts have been made to quantify differences between estimated time of arrival (ETA) and actual ambulance transport times with or without highest urgency level. Observed ambulance transport times have been compared with ETA subjectively judged by ambulance drivers [9, 10], transport time of a chase vehicle driving the same route at normal speed as an ambulance with highest urgency level [11, 12], and estimates using GIS compared to retrospective data on ambulance transports [13–15].

Google Maps is a GIS providing route-based transport time estimation and is the most used system to estimate prehospital ambulance transports in studies [3, 4, 14, 16]. The Google Maps transport estimates are influenced by several factors such as weather, time of day, and road work. In Denmark, however, the ambulances use a GIS-based commercial navigation system provided by Logis Solutions that provides ETA for the ambulance transports. Estimates of prehospital transport time are an important part of emergency medical service (EMS) planning. Nevertheless, large prospective studies comparing ambulance transport times using up-to-date GIS software are to our knowledge not performed with estimates extracted under similar conditions as the observed transports. Such a study would provide an indication of the accuracy of the GIS software used for estimating arrival time, which would benefit the reliability of simulation studies aiming to compare transport strategies [5]. In addition, the information would be valuable for planning of transports with highest urgency level and ultimately help prioritize the hands of healthcare personnel at receiving hospital departments. Finally, the study would provide an estimate of time saved using L&S in countries where such transports are allowed to break traffic rules.

Thus, we aim to quantify relative and absolute differences in medians between estimated transport times provided by Google Maps and the observed ambulance transport times from scene to hospital arrival at different levels of urgency and distances. Our primary aim is to compare transportations at highest level of urgency (Level A) with transportations at the lowest level of urgency (Level B) on the difference between GIS estimated and observed transport times.

## Methods

The study was designed as a natural experiment, since we assume that urgency level is unassociated with traffic conditions and considered random. We collected observed transport times for ambulances dispatched with urgency Level A and B from March 10 to June 11, 2021, and compared them with their corresponding estimated transport times. The estimated transport times were conditioned on corresponding weekday, time-off-day, weather conditions, and major changes to road network within 3 weeks of the observed transportation. No larger lockdowns due to the COVID-19 pandemic were started or ended within the inclusion period.

### Study population

Patient transports were included if departing by ambulance on land from private addresses throughout the Central Denmark Region and arriving at one of the region’s six EDs.

### Setting

The study was conducted in the Central Denmark Region, one of the five Danish regions, with a size of 13.011 km^2^ and a total population of 1.38 million [17, 18]. The demography of the area is mixed urban and rural with a total population density of 100.3 people per km^2^ with a span from 717.5 people per km^2^ in the biggest city of the region (Aarhus), to 8–35 people per km^2^ in rural areas [19, 20].

In the Central Denmark Region, the emergency calls from citizens are received and managed by one central Emergency Medical Dispatch Center (EMDC). Based on the symptom, believed to be most important, the EMS dispatcher allocates resources (e.g., an ambulance with or without a physician manned rapid response vehicle and/or helicopter) and determines the level of urgency using The Danish Index (DI). DI is a criteria-based dispatch protocol that consists of 37 main symptom groups (organized in chapters). Each chapter is subdivided into five levels of urgency. Three levels are dispatched for patients in need of medical treatment: Level A for life-threatening or potentially life-threatening conditions, requiring an immediate response dispatched during the emergency call conversation; Level B for urgent, but not life-threatening conditions dispatched within 30 min from the emergency call; and Level C for transport less urgent that should be dispatched within 1–2 h after the emergency call (mostly used for inter-hospital transfers and general practitioner arranged transports). Only Level A transportations uses L&S and are allowed to break traffic rules, e.g., exceeding posted speed limits, proceeding through red traffic signals, stop lights or stop signs, bypassing traffic jam, and using alternative routes, e.g., sidewalk etc. Each emergency call is assigned a DI criteria code that corresponds to the level of emergency, based on the main symptom and a specific subgroup symptom. At scene, the EMS providers assess the patient and decide whether the patient should be transported to the nearest acute medical department or specialized care. However, before departure from the scene, the EMS providers inform the receiving department, and a second opinion may be utilized. In various scenarios, ambulances may bypass nearby EDs, proceeding directly to specialized departments. For instance, local ED bypass is frequently observed in Level A transports involving patients suspected of stroke or acute myocardial infarction. Similarly, patients transported by Level B urgencies may also bypass local EDs, particularly under open admission agreements for cancer patients.

In cases where patients exhibit highly unstable vital signs, the ambulance often prioritizes proximity and heads directly to the nearest acute medical department, setting aside the bypass strategy. Consequently, the cumulative distances covered in Level A or B transports can vary based on the specific circumstances and the urgency of the medical condition.

Simultaneously, the level of urgency initially decided by the EMS dispatcher can be down or upgraded. Level of urgency from the scene to hospital can, therefore, differ from the initial dispatched urgency level.

In this study, dispatched Level A driven as Level A to hospital is noted A–A, dispatched Level A downgraded at scene and driven as Level B as A–B, and dispatched Level B driven as Level B as B–B. Transports dispatched as Level B driven as Level A were limited and could not form an independent group, thus, excluded.

### Data sources

Data from the EMDC dispatch system (Logis) and the electronic Prehospital Patients Record (ePPR) included addresses and coordinates of the scene of the accident/incident and the receiving hospital; time and date of the departure from scene and arrival at hospital; total transport duration; the Logis system’s ETA; the DI dispatch code (containing the dispatched level of urgency); and the level of urgency of the ambulance transport to hospital.

The Google Maps Application Programming Interface (API) was assessable for public use. Google Maps Distance Matrix API was used to make requests from Google Maps on the estimated transport times both non-traffic and traffic adjusted. To retrieve Google Maps estimates, we used R [R Core Team (2022). R: A language and environment for statistical computing. R Foundation for Statistical Computing, Vienna, Austria. URL https://www.R-project.org/] [21].

Addresses of all scenes of incident and receiving hospitals were converted to latitude and longitude coordinates from ePPR to provide estimated transport times in the Google Maps system. It is not possible to get estimates of historical transports in Google Maps, and thus, the corresponding estimation of duration for each transport were retrieved within 3 weeks after the transport took place conditioned on corresponding weekday, time-off-day, weather conditions, major changes to road network (e.g., road network changes, major events creating a traffic jam, etc.).

Departure time was grouped according to morning rush hour (07:00–09:00), daytime (09:00–15:00), afternoon rush hour (15:00–17:00), and nighttime (17:00–07:00). The mode was set to ‘*driving*’ and traffic-model was set to ‘*best guess*’, which was the best estimate of travel time given what was known about both historical traffic conditions and live traffic information*.*

We retrieved data on the daily weather conditions from the Danish Meteorological Institute (DMI). Weather conditions were only grouped according to above or below 2 ℃. However, transports that took place on days with reports of snow, heavy rain (defined as more than 24 mm within 6 h), and heavy fog (defined as visibility less than 100 m) were excluded. Transportations where it was impossible to find an estimated transportation duration corresponding to the stated conditions were also excluded.

### Outcome

The outcome was transport duration from scene of accident to receiving hospital department.

### Statistical analysis

Initially, we summarized the distribution of urgency level among indications for emergency transport using the DI. Distributions were presented as frequencies and percentages, and indications of emergency transports which represented less than 100 transports were discarded.

In the primary analysis, we estimated absolute and relative differences in medians between observed and estimated transport durations. These were stratified by Level A–A, A–B, and B–B. Results were calculated as duration of the entire transport and per 10 km. Standard errors were estimated using bootstrap with 1000 replications. In addition, results were stratified by distances below 10 km, from 10 to 20 km, and above 20 km.

We conducted a sensitivity analysis where we used the estimated transport times provided by Logis instead of Google maps.

Finally, using restricted cubic splines, we conducted median regression spline curve analysis on transports less than 100 km to visualize duration as a function of distance comparing Level A–A and B–B transports with Google Maps estimated durations. From the spline curves, we were able to estimate the median delay from the ambulance personnel activated Logis and until the ambulance left the scene. This delay was estimated by subtracting the intercept of the spline curves for the observed transport durations with the intercept of the spline curves for the estimated durations.

Results were presented with 95% confidence intervals (CI) and data were analyzed using Stata 17 (StataCorp. 2021. Stata Statistical Software: Release 17. College Station, TX: StataCorp LLC.).

## Results

**Fig. 1:**
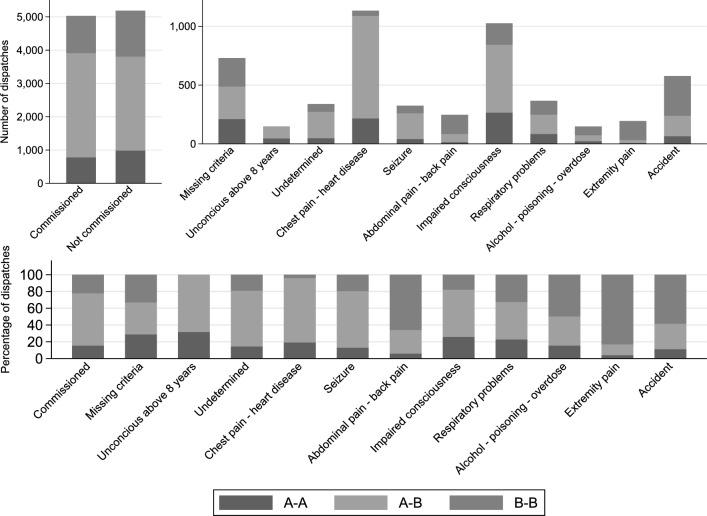
Distribution of urgency level among indications for emergency transport as reported by the emergency medical service (EMS) dispatcher. Top left: absolute numbers of commissioned and not commissioned transports. Top right: absolute numbers of the most common emergency indications among non-commissioned transports. Bottom: Relative distribution of all indications for emergency transport including commissioned transport

In the study period, a total of 12,434 ambulance transports were dispatched with an urgency level of either A or B. After exclusion, the study population consisted of 10,939 ambulance transports with 1981 Level A–A, 6228 Level A–B, and 2730 Level B–B transports (see Figure S1 for details). For Level A–A transports, 25.6% (*n* = 699) left the scene during weekend and 62.1% (*n* = 1696) left the scene between 6 AM and 6 PM. This distribution was 25.6% (*n* = 1597) and 64.9% (*n* = 4042) for Level A–B, and 28.5% (*n* = 565) and 62.3% (*n* = 1235) for Level B–B. Distribution of urgency level on the six EDs and acute stroke center stratified by distance from scene to hospital is shown in Table S1. The distribution of urgency level among indications for emergency transport is shown in Fig. 1.

The median distance for Level A–A transports was 22.6 km (IQR 12.6; 33.1), Level A–B transports 22.4 km (IQR 12.4; 36.3), and Level B–B transports 24.6 km (IQR 13.0; 38.4). Median observed durations per 10 km for each urgency level are shown in Table 1.

**Table 1:** Median transport time (minutes per 10 km) with interquartile ranges for observed transports to hospital

Dispatch groups	Transports up to 10 km	Transports 10–20 km	Transports above 20 km	All distances
A–A	15.6 (12.5; 25.4)	9.5 (8.2; 11.7)	7.1 (6.0; 8.9)	7.9 (6.2; 11.9)
A–B	24.6 (19.4; 35.1)	13.7 (11.3; 17.1)	10.4 (8.8; 12.8)	12.9 (9.6; 22.0)
B–B	25.3 (19.8; 37.8)	14.4 (11.8; 19.1)	10.4 (8.9; 13.1)	13.1 (9.6; 22.2)

Dispatch groups = first letter is dispatched level of urgency, and second letter is level of urgency for the actual transport to hospital

Table 2 shows the absolute and relative differences between estimated and observed transport durations per 10 km, while Table 3 shows the absolute and relative differences between estimated and observed transport durations for the actual duration. Both tables are stratified according to distance and urgency level. The tables also depict the sensitivity analysis. In all analyses, Google maps significantly overestimated the duration for urgency Level A–A, while they significantly underestimated the transport duration for urgency Level A–B and B–B. For Level A–A, the relative difference between estimated and observed was quite similar across all distances per km (Table 2) as well as for the actual duration (Table 3). However, for Level A–B and B–B, the relative difference between estimated and observed was reduced with longer distances (Tables 2, 3). Our sensitivity analysis shows that Logis also significantly overestimated the duration for urgency Level A–A, while they significantly underestimated the transport duration for urgency Level A–B and B–B. However, indirectly comparing Logis and Google maps estimation of duration, indicates that Logis estimates transports to be shorter in time per km than the estimates provided by Google maps on the transport below 10 km (Table 2). On longer distances, the difference per 10 km between Logis and Google maps diminished. However, for the total duration of the transport, Logis again estimated transports to be significantly shorter in time than the estimates provided by Google maps on the longest transports (Table 3).

**Table 2:** Absolute (minutes) and relative (%) differences between median estimated and median observed transport times per 10 km across urgency levels

Level		Transports up to 10 km	Transports 10–20 km	Transports above 20 km	All distances
Median Google Maps estimated vs median observed transport times per 10 km					
A–A	Abs, min (95% CI)	4.8 (3.9; 5.6)	2.9 (2.5; 3.4)	2.1 (2.0; 2.3)	1.9 (1.8; 2.0)
	Rel, % (95% CI)	30.4 (23.4; 37.4)	31.0 (25.8; 36.3)	32.4 (30.4; 34.4)	24.3 (22.5; 26.1)
A–B	Abs, min (95% CI)	−4.0 (−4.5; −3.6)	−1.4 (−1.7; −1.2)	−0.5 (−0.6; −0.4)	−1.5 (−1.7; −1.3)
	Rel, % (95% CI)	−16.3 (−18.0; −14.7)	−10.6 (−12.4; −8.8)	−5.1 (−5.8; −4.4)	−11.8 (−13.0; −10.5)
B–B	Abs, min (95% CI)	−4.4 (−5.4; −3.5)	−2.0 (−2.4; −1.6)	−0.5 (−0.7; −0.4)	−1.8 (−2.1; −1.5)
	Rel, % (95% CI)	−17.5 (−20.6; −14.5)	−14.0 (−16.8; −11.2)	−5.5 (−6.6; −4.3)	−13.7 (−15.7; −11.7)
Median Logis estimated vs median observed transport times per 10 km					
A–A	Abs, min (95% CI)	3.1 (2.3; 3.9)	3.1 (2.8; 3.4)	2.0 (1.9; 2.1)	2.0 (1.9; 2.2)
	Rel, % (95% CI)	19.8 (14.1; 25.5)	32.7 (28.5; 36.9)	30.8 (28.8; 32.8)	25.7 (23.7; 27.7)
A–B	Abs, min (95% CI)	−5.3 (−5.8; −4.9)	−1.2 (−1.4; −0.9)	−0.5 (−0.6; −0.4)	−1.3 (−1.5; −1.1)
	Rel, % (95% CI)	−21.7 (−23.1; −20.3)	−8.5 (−10.3; −6.7)	−5.4 (−6.2; −4.6)	−10.3 (−11.6; −9.0)
B–B	Abs, min (95% CI)	−6.4 (−7.3; −5.5)	−2.0 (−2.4; −1.7)	−0.6 (−0.7; −0.4)	−1.7 (−2.0; −1.5)
	Rel, % (95% CI)	−25.2 (−28.0; −22.4)	−14.2 (−16.4; −12.1)	−5.9 (−7.1; −4.6)	−13.3 (−15.3; −11.4)

Estimates are shown for all distances and stratified by transport distances of < 10 km, 10–20 km and > 20 km. *A–A* dispatch as Level A and transported to hospital as Level A, *A–B* dispatched as Level A and transported to hospital as Level B, *B–B* dispatched as Level B and transported to hospital as Level B, *CI* confidence interval, *Abs* absolute time difference, *rel* relative time difference, *min* minutes

**Table 3:** Absolute (minutes) and relative (%) differences between median estimated and median observed transport times across urgency levels

Level		Transports up to 10 km	Transports 10–20 km	Transports above 20 km	All distances
Median Google Maps estimated vs median observed transport times					
A–A	Abs, min (95% CI)	2.4 (1.8; 2.9)	4.7 (4.1; 5.3)	8.6 (7.7; 9.5)	6.2 (5.4; 6.9)
	Rel, % (95% CI)	30.8 (23.0; 38.6)	33.8 (28.8; 38.9)	29.9 (26.5; 33.3)	27.3 (23.8; 30.7)
A–B	Abs, min (95% CI)	−2.1 (−2.4; −1.9)	−1.5 (−1.9; −1.1)	−1.1 (−1.6; −0.7)	−1.5 (−1.9; −1.0)
	Rel, % (95% CI)	−19.8 (−22.0; −17.5)	−7.6 (−9.5; −5.6)	−3.2 (−4.5; −1.9)	−6.6 (−8.5; −4.6)
B–B	Abs, min (95% CI)	−2.4 (−2.9; −1.8)	−2.5 (−3.2; −1.8)	−1.7 (−2.5; −0.9)	−2.8 (−3.6; −1.9)
	Rel, % (95% CI)	−21.2 (−25.4; −17.0)	−11.7 (−14.6; −8.7)	−4.7 (−6.8; −2.5)	−11.2 (−14.5; −8.0)
Median Logis estimated vs median observed transport times					
A–A	Abs, min (95% CI)	1.8 (1.3; 2.2)	4.1 (3.5; 4.7)	7.2 (6.3; 8.0)	5.2 (4.5; 5.8)
	Rel, % (95% CI)	22.8 (16.7; 28.8)	29.4 (24.6; 34.3)	24.8 (21.7; 27.9)	22.8 (19.8; 25.9)
A–B	Abs, min (95% CI)	−2.4 (−2.6; −2.1)	−2.0 (−2.3; −1.6)	−2.8 (−3.2; −2.4)	−3.1 (−3.5; −2.7)
	Rel, % (95% CI)	−22.1 (−23.9; −20.2)	−9.8 (−11.6; −8.0)	−7.9 (−9.1; −6.8)	−13.8 (−15.5; −12.1)
B–B	Abs, min (95% CI)	−2.6 (−3.2; −2.1)	−3.3 (−4.0; −2.5)	−3.4 (−4.2; −2.7)	−4.7 (−5.4; −4.0)
	Rel, % (95% CI)	−23.8 (−27.5; −20.1)	−15.4 (−18.3; −12.5)	−9.4 (−11.3; −7.6)	−19.1 (−21.8; −16.4)

Estimates are shown for all distances and stratified by transport distances of < 10 km, 10–20 km, and > 20 km. *A–A* dispatch as Level A and transported to hospital as Level A, *A–B* dispatched as Level A and transported to hospital as Level B, *B–B* dispatched as Level B and transported to hospital as Level B, *CI* confidence interval, *Abs* absolute time difference, *rel* relative time difference, *min* minutes

Based on the results in Table 1, the median duration per km of transports with urgency Level B–B was 66.2% (95% CI 59.6; 72.8) longer than transports with urgency Level A–A. For distances less than 10 km, the difference was 61.6% (95% CI 50.7; 72.4), while the difference reduced to 52.0% (95% CI 45.4; 58.6) and 48.3% (95% CI 45.3; 51.4) for distances between 10 and 20 km and above 20 km, respectively.

**Fig. 2:**
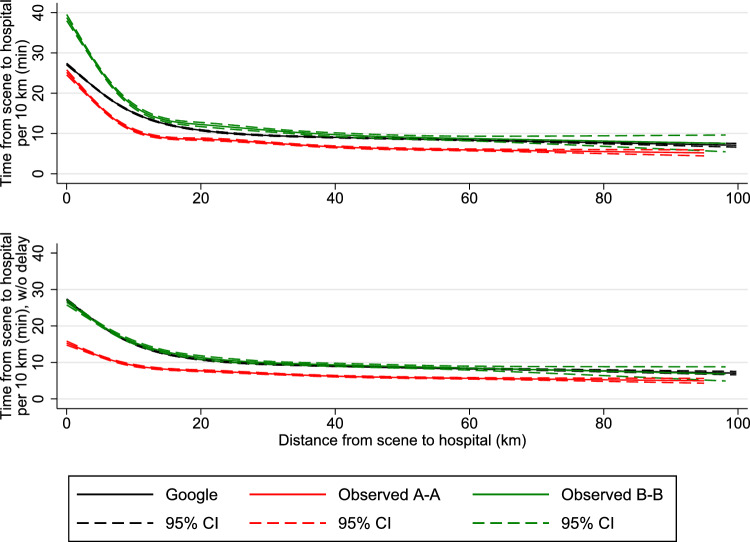
Observed and Google estimated transports plotted according to distances driven (*x*-axis) and time duration per 10 km (*y*-axis). Google estimates are based on the transport coordinates from the observed A–A and B–B transports. A–A = dispatched as Level A and transported to hospital as Level A, B–B = dispatched as Level B and transported to hospital as Level B, line (black, green, and red) = median transport time for all distances. CI = confidence intervals. Transports are restricted to distances below 100 km. Bottom figure: Same as top figure, but with a delay of 1.8 min subtracted from all observed transport times. See Figure S2 for the graphs of the top figure combined with scatter plots

**Fig. 3:**
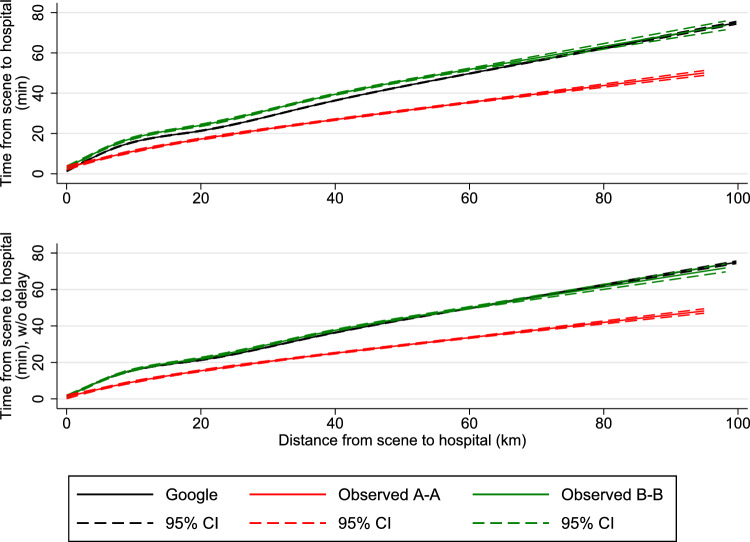
Observed and Google estimated transports plotted according to distances driven (*x*-axis) and time duration of the entire journey (*y*-axis). Google estimates are based on the transport coordinates from the observed A–A and B–B transports. A–A = dispatched as Level A and transported to hospital as Level A, B–B = dispatched as Level B and transported to hospital as Level B, line (black, green, and red) = median transport time for all distances. CI = confidence intervals. Transports are restricted to distances below 100 km. Bottom figure: Same as top figure, but with a delay of 1.8 min subtracted from all observed transport times

Using the spline curve analysis for the observed and estimated transports, the median delay from the EMS providers activated Logis and until the ambulance left the scene was 1.5 min (95% CI 0.5; 2.6) for urgency Level A–A and 1.6 min (95% CI 0.7; 2.4) for urgency Level B–B. For the combined spline curve of urgency Level A–A and B–B, the median delay was 1.8 min (95% CI 1.2; 2.5). Figures 2 and 3 show the estimated spline curves for transport durations per 10 km and observed transport time, respectively. In both figures, the top graphs depict the observed transport durations, while in the bottom figures, all observed transport times subtracted 1.8 min. In the bottom graphs, the Google Maps estimated median durations almost completely overlap the observed transport times for urgency Level B–B, which emphasizes the accuracy of Google Maps, when accounting for the delay.

## Discussion

To our knowledge, this is the first study that compares transport duration between highest and lowest ambulance urgency level relative to the Google Maps estimation in a setting where ambulances are allowed to break normal traffic rules. Our spline curve analyses show that the transports with highest urgency level are significantly superior to transports with lowest urgency level at all distances. Time saved using highest urgency level of urgency is inversely proportional to the distance driven. This is supported by our main findings which shows differences per 10 km between urgency Level A and B transports of more than nine-minute for transports of less than 10 km, 4 min for transports between 10 and 20 km and 3 min for transports above 20 km. Compared with Google Maps estimates, a relative constant proportional difference of 30% was seen in favor of Level A transports at all distances (< 10 km, 10–20 km and > 20 km). In all ambulance transports (Level A and B), a small but consistent delay was observed from activation of the ambulance navigation system to actual driving probably illustrating a need of time for all passengers including the patient to be fastened before transport. When this initial delay was subtracted, all Level B transport durations were equal to the Google maps estimations (Figs. 2, 3). Logis consequently estimated transport times to be shorter than Google Maps but not more accurately.

We found a difference in median transport distance between urgency Level A and B, with Level B transport being 2 km longer. It is likely that transports with urgency Level B bypass the local centers and drives directly to a specialized care center more often than transports with urgency Level A, which would explain the difference in median travel distance. Moreover, a large portion of commissioned transports were classified as urgency Level A–A or A–B. Many of these transports are likely commissioned by a general practitioner, in which case the indication of emergency is not recorded in the available records and instead classified as commissioned.

Using L&S and allowing traffic laws to be broken substantially saves time. A resource consuming setup was used in a small study (*n* = 67) from the United States, where a “chase” ambulance (without a patient) subsequently drove the same route as an on-duty ambulance with the highest urgency level. A 30.9% reduction in transport time was found in favor of transports with the highest level of urgency [11]. This equals the results from our study when transports with the highest level of urgency were comparing with the Google Maps estimates. Using a “chase” ambulance, the study did not account for any delay related to driving an ambulance with a real patient and with attending EMS providers which makes the results comparable to the Google Maps estimates but not real Level B ambulance transports.

With the high number of prospectively included transports in our study, it was possible to calculate differences for all distances driven and to adjust for weekday, time of day, weather, and major traffic obstructions, which was not possible in studies using retrospective data [13–15].

Published but non-peer-reviewed data on 5000 ambulance transports at highest urgency level from London [21] showed a similar difference between Google estimates and observed ambulance transports as found in our study, hence also equal to the difference between ambulance transports with highest level of urgency and the “chase” ambulance in the US study by Ho and Lindquist [11]. In the London data, most transport durations were less than 15 min, and the ambulances were found to be 1.355 times quicker than regular road traffic for transports less than 10 km. It is noticeable that the results from London as an urban area translate to our mixed urban and rural setting for ambulance transports < 10 km and it seems to indicate that cities of different sizes influence equally on the proportional difference between observed transport times and estimates from Google. Also, it points to a universal and fixed correction factor, at least for transports < 10 km or with a duration up to 15 min. Using Google Maps estimations as reference, ambulance transports with highest level of urgency might be predicted with high accuracy in urban and mixed urban and rural areas without the need of collecting own transport data to improve accuracy as suggested by many [14, 15, 22, 23].

Only when using the highest level of urgency, the ambulances can use L&S which allows them to break normal traffic rules carrying an increased risk of accidents [24, 25]. Based on US data, benefit on patient outcome has not been shown from using highest level of urgency [26]. However, very large proportions (> 80%) of all transports in the US are performed at highest level of urgency [27, 28]; this compared to < 20% in our study (Figure S1). The highest level of urgency greatly reduces transport time, and with a medically prioritized transport strategy, it is believed to be of vital importance for conditions as cardiac arrest, chest pain, severe trauma, severe respiratory difficulties, and stroke [29–33]. In Denmark, stroke alone accounted approximately 560 acute admissions per 100.000 in 2010–2012 [34]. A study by Meretoja et al. showed that 15 min saved in the ambulance translates to an additional month of healthy life on average [31]. On the negative side, a study from the US showed that the crash rate increased from 7.0 of 100,000 when transporting without L&S to 17.1 of 100,000 with L&S. However, for the yearly number of acute stroke patients in Denmark, which currently accounts a population of 5.9 million, the increase in crash rate affects less than one patient [18]. Thus, our results may affect policymaking in countries where ambulances are not allowed to break traffic rules even when transporting at highest urgency level. Moreover, in prehospital health care planning or health care facilities using GIS-based ETAs to estimate ambulance transport times, the differences in transport times between different levels of urgency must be incorporated to reduce valuable waiting time at the receiving hospital departments.

A limitation to this study was the chosen time-period. We deliberately avoided cold days with snowy or icy roads, because the same conditions should be matchable for the Google estimates on a subsequent day within the 3-week-period from the observed ambulance transport. It would have been ideal to include a full year of ambulance journeys and to match Google Maps estimates on the precise time of the observed transports. A seasonal bias from this study could exist. The study was only conducted in one of Denmark’s five regions the Central Denmark Region and the study might lose some external validity on that account. However, the study is strengthened by the sizable sample of records gathered from electronic records including coordinates of scene of accident and receiving hospital departments.

In conclusion, ambulance transport times are greatly reduced when using highest level of urgency.

A universal and fixed proportional difference of 30% seems to exist between Google Maps estimates and actual ambulance transports with highest level of urgency irrespective of city size and this difference must be accounted for when using a GIS-based systems for ETA. A small but consistent delay before initiation of the ambulance transports must be added to make Google Maps estimates reflect ambulance transports of lowest level of urgency.

### Supplementary Information

Below is the link to the electronic supplementary material.

Supplementary file1 (PDF 459 KB)

## Data Availability

Data can be shared on reasonable requests and in accordance with Danish legislation.

## Abbreviations

API: Application Programming Interface
DI: Danish Index
ED: Emergency department
EMDC: Emergency Medical Dispatch Center
EMS: Emergency medical service
ePPR: Electronic Prehospital Patients Record
ETA: Estimated time of arrival
L&S: Lights and sirens
