# Supplementary material for: Effect of urgency level on prehospital emergency transport times: a natural experiment
Source: Intern Emerg Med. 2023 Dec 20;19(2):445–53. doi: 10.1007/s11739-023-03501-7 (PMC10954969; doi:10.1007/s11739-023-03501-7)

Supplemental material

Figure S1: Flow chart. Ambulance transports.

A-A = dispatched and transported to hospital as Level A;

A-B = dispatched as Level A but transported as Level B;

B-B = dispatched and transported as Level B.

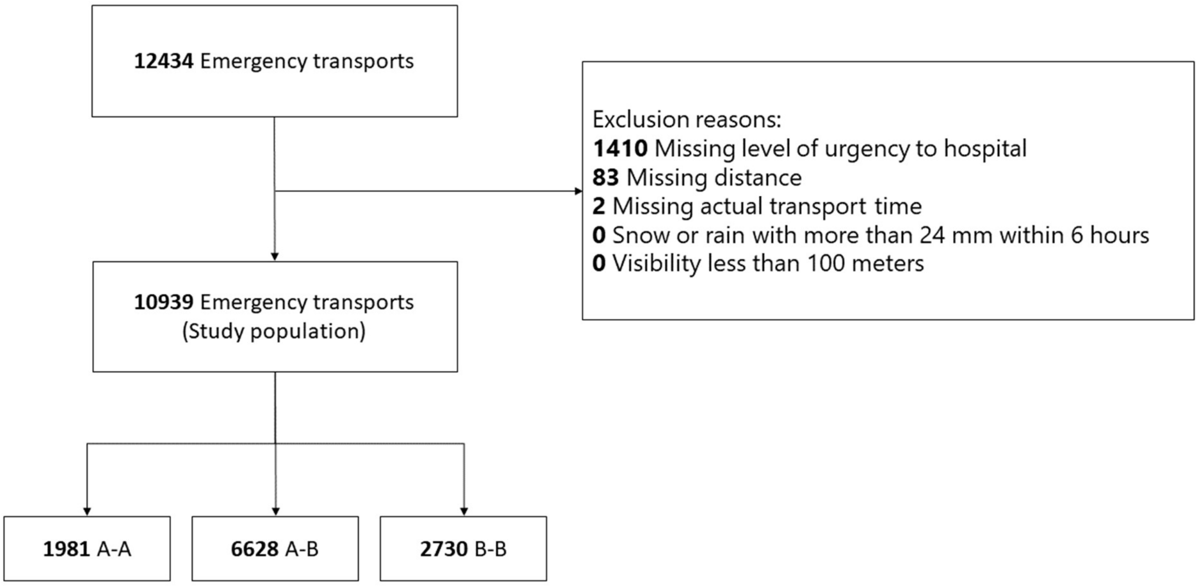

Table S1. Distribution of ambulance transports to an emergency department or acute stroke center.

Hospital	A-A<br>n = 1981	A-B<br>n = 6228	B-B<br>n = 2730	Total<br>n = 10.939
Aarhus University Hospital % (n)	45.0 (892)	28.5 (1772)	28.4 (776)	31.4 (3440)
Herning Regional Hospital % (n)	15.2 (301)	18.4 (1145)	16.4 (449)	17.3 (1895)
Randers Regional Hospital % (n)	12.6 (249)	16.0 (996)	16.1 (440)	15.4 (1685)
Horsens Regional Hospital % (n)	9.4 (187)	15.6 (969)	14.5 (396)	14.2 (1552)
Viborg Regional Hospital % (n)	9.4 (186)	15.2 (945)	17.3 (471)	14.6 (1602)
Holstebro Regional Hospital % (n)	8.4 (166)	6.4 (401)	7.3 (198)	7.0 (765)

Figure S2: Observed and Google estimated transport time per 10 km plotted according to distances driven (x-axis) and time duration (y-axis). Google estimates are based on the transport coordinates from the observed A-A and B-B transports. A-A = dispatch as level A and transported to hospital as level A, B-B = dispatched as level B and transported to hospital as level B, solid line (black, green and red) = median transport time for all distances.

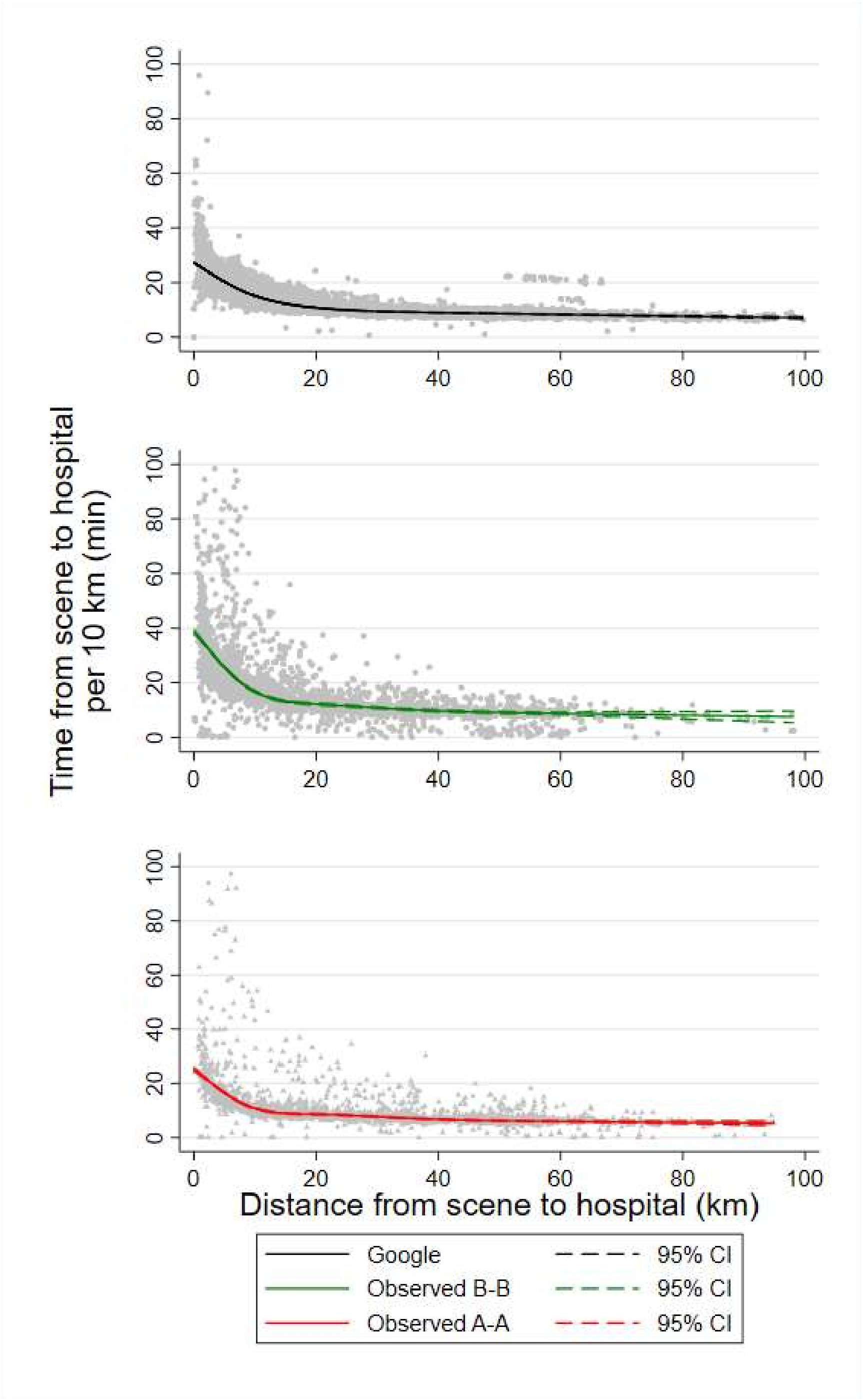

Supplement: Supplementary file 1 — Supplementary file1 (PDF 459 KB) [file 11739_2023_3501_MOESM1_ESM.pdf]
